# Alterations in the gut microbiota of alcoholic cirrhosis patients infected with *Clonorchis sinensis* in the Pearl River Delta region of China

**DOI:** 10.1371/journal.pone.0334311

**Published:** 2025-10-21

**Authors:** Aijun Huang, Minxuan Su, Shebin Zhang, Chanjing Zhao, Yanfen Luo, Yifei Long, Dongling Lin, Song Li, Cong Shen, Qiwei Li, Yimei Cai, Lina Wang, Jianping Liu, Cha Chen

**Affiliations:** 1 Department of Laboratory Medicine, The Second Affiliated Hospital of Guangzhou University of Chinese Medicine, Guangzhou, China; 2 The second Clinical Medical College, Guangzhou University of Chinese Medicine, Guangzhou, China; Institute of Cytology and Genetics SB RAS: FIC Institut citologii i genetiki Sibirskogo otdelenia Rossijskoj akademii nauk, RUSSIAN FEDERATION

## Abstract

Clonorchiasis, a foodborne parasitic disease caused by *Clonorchis sinensis* (*C. sinensis*), is prevalent in certain regions of Asia and can result in severe hepatobiliary complications, including cholangiocarcinoma, peribiliary fibrosis, and hepatic fibrosis. In certain regions of China, the concurrent consumption of raw freshwater fish and alcohol, which are components of the local dietary culture, has contributed to the high prevalence of this disease. Infected individuals often endure the dual burden of clonorchiasis and alcoholic liver disease (ALD). While both *C. sinensis* infection and alcohol abuse can disrupt the gut microbiota, the synergistic mechanisms of these two factors in patients with alcoholic cirrhosis (ALC) remain poorly understood. This study aims to elucidate the impact of *C. sinensis* infection on the gut microbiota of patients with ALC, with the objective of identifying potential diagnostic or therapeutic targets. A total of 64 patients diagnosed with ALC were recruited for this study, with half of the participants infected with *C. sinensis* and the other half remaining uninfected. Fresh fecal samples were collected from all participants. Alterations in the gut microbiota were analyzed using high-throughput sequencing of the 16S ribosomal RNA gene derived from the fecal samples. Our results showed that analysis of β-diversity revealed significant differences in gut microbial communities between *C. sinensis*-infected and non-infected groups (*P* < 0.05). The Linear discriminant analysis effect size (LEfSe) identified the phylum Firmicutes (highest LDA score at the phylum level) and the genus *Prevotella* (dominant at the genus level) as key taxa driving gut microbial composition differences in patients with ALC and *C. sinensis* infection (*P* < 0.05). Correlation analysis at the genus level demonstrated significant negative associations between *Enterococcus* and multiple bacterial genera in the *C. sinensis*-infected group. Kyoto Encyclopedia of Genes and Genomes (KEGG) pathway analysis further highlighted divergent metabolic pathways between groups, with the vancomycin resistance pathway showing the most pronounced disparity. In conclusion, patients with ALC and *C. sinensis* infection show alterations in gut microbiota compared to non-infected counterparts. Notably, specific bacteria such as *Prevotella* and *Enterococcus* may represent potential targets for the development of novel diagnostic and therapeutic strategies for ALC patients afflicted by *C. sinensis* infection.

## Introduction

Clonorchiasis is an important foodborne parasitic disease primarily acquired through the consumption of raw or undercooked infected freshwater fish and shrimp. It is closely associated with various severe complications of the hepatobiliary system, including cholangiocarcinoma, peribiliary fibrosis, and hepatic fibrosis [1–3]. *Clonorchis sinensis* (*C. sinensis*) is primarily prevalent in Asia, including China, South Korea, northern Vietnam, and parts of Russia, with an estimated global infection rate of 15 million people [4–9]. In China, clonorchiasis is a regional parasitic disease, with high infection rates in certain areas [10]. According to the latest (third) national survey on the current status of key human parasitic diseases in China, as of 2015, approximately 5.98 million individuals were infected with *C. sinensis* nationwide. Notably, in urban and peri-urban areas of the Pearl River Delta, the infection rate reached a significant prevalence of 23.36% [11]. The consumption of “yu sheng,” a raw fish delicacy, remains prevalent as both a culinary specialty and a cultural tradition in numerous counties and municipalities across Guangdong Province and the Guangxi Zhuang Autonomous Region. Notably, the practice of consuming raw freshwater fish has been systematically documented as an endemic dietary behavior since the Qing Dynasty (1644–1912) [11–13].

Meanwhile, the culture of consuming Chinese liquor is deeply ingrained in China, particularly among men, where drinking etiquette and customs are regarded as essential means of fostering social interaction and trust [14–16]. In regions where *C. sinensis* is endemic, susceptible populations frequently consume alcohol while eating uncooked freshwater fish. This behavior is influenced both by the prevailing drinking culture and by the belief that alcohol can eliminate the metacercariae of *C. sinensis* present in raw fish [1, 2]. Consequently, the local population often experiences a dual burden of clonorchiasis and alcoholic liver disease (ALD).

ALD can be classified as mild alcoholic liver disease, alcoholic hepatic steatosis, alcoholic hepatitis, alcoholic liver fibrosis, and alcoholic cirrhosis (ALC) [17]. Approximately 8% −20% of patients with ALD progress to ALC [18]. Explosive economic growth and increasing social openness in China over the last 30 years have significantly boosted alcohol consumption, and consequently, the incidence of ALD in China has increased [19]. ALD is closely associated with the gut microbiota. Existing studies have established that the gut microbiota has been identified as a key player in the severity of liver injury in ALD [20]. Dysbiosis of the gut microbiota contributes to individual susceptibility to ALD [21], and further facilitate its development and progression [22].

Moreover, there exists a highly intricate system of interaction between parasites and microbial communities [23]. The microbiota can influence the survival, reproduction, and virulence of parasites, while parasites can effectively reduce or increase the diversity of microbial species [24]. Existing studies have confirmed that *C. sinensis* infection can alter the host’s immune system and induce changes in the composition of the gut microbiota [25–27]. However, studies on the gut microbiota in patients with ALC and *C. sinensis* infection remain limited. Given the strong association between *C. sinensis* infection and alcohol consumption in China [2], this study is the first to investigate the impact of *C. sinensis* infection on the gut microbial composition of ALC patients, filling the gap in research on *C. sinensis* infection within this population. Our findings may provide potential diagnostic and therapeutic targets for ALC complicated by *C. sinensis* infection.

## Methods

### Subject recruitment

A total of 201 patients diagnosed with ALC at four branches of the Guangdong Provincial Hospital of Traditional Chinese Medicine were screened according to the established exclusion criteria between January 2023 and July 2024. Subsequently, 64 adult patients diagnosed with ALC were recruited, comprising 32 patients combined with *C. sinensis* infection (3 females and 29 males) and 32 non-infected patients (5 females and 27 males). This study was conducted in accordance with the Declaration of Helsinki and received approval from the Ethics Committee of Guangdong Provincial Hospital of Traditional Chinese Medicine (ZE2022-245-01). Written informed consent was obtained from all participants.

**Inclusion criteria**

1 The diagnostic criteria for ALC adhere to Guidelines of prevention and treatment for ALD (2018, China) [28]as well as Chinese consensus on the management of liver cirrhosis [29]:
  ① Participants with a history of alcohol consumption ≥5 years, equivalent to an ethanol intake of ≥40 g/day for males and ≥20 g/day for females; or a history of excessive alcohol consumption (>80 g ethanol/day) within the preceding 2 weeks. The ethanol amount (g) was calculated using the formula: Alcohol volume (mL) × Ethanol concentration (%) × 0.8.
  ② Histopathological findings were consistent with the diagnosis of cirrhosis.
  ③ Endoscopic examination revealed esophagogastric varices or ectopic varices, excluding non-cirrhotic portal hypertension.
  ④ Imaging examinations such as ultrasonography, liver stiffness measurement (LSM), CT, and MRI indicate typical features of cirrhosis or portal hypertension (the diagnostic threshold of LSM for ALC is 20 kPa).

On the basis of excluding other causes of liver disease such as viral hepatitis infection, drug-induced or toxic liver injury, and autoimmune liver disease, a diagnosis of ALC can be made if the patient meets criterion ① and any one of criteria ②, ③, or ④.

2 Diagnosis of *C. sinensis* Infection: Infection can be confirmed by detecting *C. sinensis* eggs in the patient’s fecal samples. Initially, fecal samples are collected from the patient and processed using the ORIENTER Automatic Feces Processing Analyzer FA280 to screen for positive samples of *C. sinensis* eggs. Subsequently, the modified Kato-Katz thick smear method is used for re-examination. The detection of *C. sinensis* eggs through microscopic examination confirms the positive result.

**Exclusion criteria**

- Patients under the age of 18 years and those with incomplete clinical information;
- Pregnant women;
- Patients with liver disease caused by viral hepatitis infection, drug-induced or toxic liver injury, autoimmune liver disease, or other etiologies;
- Patients with liver cancer;
- Patients who have undergone liver transplantation;
- Patients whose feces show other types of parasite eggs;
- Patients who have used antibiotics within the past three months;
- Patients with malignant intestinal tumors, those who have undergone partial intestinal resection, or those with other severe gastrointestinal diseases.

After strict selection based on inclusion and exclusion criteria, the final 64 patients included in the study were all Han Chinese, residing in the Pearl River Delta region of China. They shared similar geographical and dietary backgrounds and had no unique dietary habits.

### Sample collection

Fresh feces were sampled in a clean environment using a disposable sampling spoon and placed in a 5mL sterile sampling tube, then promptly frozen in a −80°C refrigerator. Five milliliters of fasting venous whole blood were collected from each patient for the first time after enrollment. Serum was obtained by centrifugation for biochemical indicator testing.

### DNA extraction and PCR amplification process

Genomic DNA was extracted from the samples using the cetyltrimethylammonium bromide (CTAB) method. The integrity of the DNA was assessed via 1% agarose gel electrophoresis, while the concentration and purity were determined using spectrophotometers. An appropriate amount of the DNA sample was transferred to a centrifuge tube and adjusted to a concentration of 1 ng/μL with sterile water. PCR amplification of the V3–V4 hypervariable regions of the 16S rRNA gene was performed using the universal primers. The amplification program was as follows:

Template DNA: Genomic DNA diluted to 1 ng/μL (5 μL, total 5 ng), verified for integrity via electrophoresis and spectrophotometry (A_260_/A_280_ = 1.8–2.0).

Primers: 341F (5’-CCTACGGGNGGCWGCAG-3’) and 806R (5’-GGACTACHVGGGTWTCTAAT-3’).

**Reaction System (30 μL)**:

- Phusion® High-Fidelity PCR Master Mix with GC Buffer (2×): 15 μL;
- Forward Primer (15 μM): 1 μL;
- Reverse Primer (15 μM): 1 μL;
- gDNA (1 ng/μL): 5 μL;
- Nuclease-free H_2_O: 8 μL.

**PCR Program (Bio-Rad T100)**:

① Initial Denaturation: 98 °C, 1 min.
② 30 Cycles:

- Denaturation: 98 °C, 10 s;
- Annealing: 55 °C, 30 s (optimized via gradient PCR);
- Extension: 72 °C, 45 s.

③ Final Extension: 72 °C, 5 min

### PCR product purification and normalization

PCR products were purified using AMPure XP beads (0.8 × bead volume) to remove primer dimers and short fragments. The purified DNA was eluted in 0.1 × TE Buffer (pH 8.0), and its concentration was measured using a Qubit 4.0 fluorometer (dsDNA HS Assay, Thermo Fisher). Samples were normalized to equimolar concentrations based on their DNA concentrations and the average fragment length (600 bp).

### Library construction and sequencing

Libraries were constructed using the NEBNext® Ultra II DNA Library Prep Kit (New England Biolabs) following the manufacturer’s protocol. The final libraries were assessed for size distribution (250–700 bp) using an Agilent 2100 Bioanalyzer (High Sensitivity DNA Kit) and quantified by qPCR with the KAPA Library Quantification Kit (Roche). Qualified libraries were pooled equimolarly and sequenced on the Illumina NovaSeq 6000 platform with 2 × 250 bp paired-end reads.

### Sequence analysis

1. Quality filtering: Raw reads obtained from sequencing were first filtered using Trimmomatic (v0.33). Then, cutadapt (v1.9.1) was used to identify and remove primer sequences, resulting in clean reads without primer sequences.
2. Paired-end read merging: Clean reads from each sample were merged using Usearch (v10) based on overlaps. Length filtering of the merged data was performed according to the length range specific to the target region.
3. Chimera removal: Chimeric sequences were detected and removed using UCHIME (v4.2) to obtain final effective reads.
4. OTU Clustering: Effective reads were clustered into Operational Taxonomic Units (OTUs) at a 97.0% [30] similarity threshold using Usearch (v10).

### Bioinformatics and statistical analysis

Statistical analysis of patients’ clinical biochemical parameters and demographic characteristics was performed using SPSS (v27.0.1). Taxonomic annotation of feature sequences was conducted using the Naive Bayes Classifier with alignment against the SILVA reference database. Sequencing data were primarily analyzed using the Quantitative Insights into Microbial Ecology 2 (QIIME2, v2024.5) and R packages (v4.4.1), including vegan and picante for diversity analyses, ggplot2 and VennDiagram for visualization, Tax4Fun2 for functional prediction, as well as stats for statistical testing. Rarefaction curves were generated to assess whether the sequencing depth adequately captured species diversity and indirectly reflected species richness [31].The Chao1 and Shannon indices were used to demonstrate the α-diversity of the samples, while β-diversity was quantified using Non-metric Multidimensional Scaling (NMDS) and Principal Coordinates Analysis (PCoA). To identify microbial communities at several taxonomic levels, Linear discriminant analysis effect size (LEfSe) was used to find differentially abundant taxa between groups, and the cutoff logarithmic linear discriminant analysis (LDA) score was set at 3.0. A circular network diagram based on Spearman correlation coefficients (threshold: 0.1) was constructed to visualize genus-level correlations between microbial communities in the two groups. Tax4Fun2 was employed to perform taxonomic classification of 16S high-throughput sequencing data through the QIIME2 platform using the SILVA database. Based on the classification results, 16S rRNA gene copy numbers were normalized applying NCBI genomic annotations. Finally, by establishing a mapping relationship between the SILVA taxonomic system and prokaryotic classifications in the KEGG database, KEGG functional prediction for prokaryotic microbial communities was achieved. Statistical analyses were performed using Fisher’s exact test, Student’s t-test, Mann-Whitney U test, ANOSIM, PERMANOVA, and Kruskal-Wallis rank-sum test, depending on data types and analytical objectives. *P* < 0.05 was considered statistically significant.

## Results

### Characteristics of patients

The demographic and clinical characteristics of all patients are shown in **Table 1**. There were no statistically significant variations with gender, age, leucine aminopeptidase (LAP), alanine aminotransferase (ALT), total protein (TP), alkaline phosphatase (ALP), γ-glutamyltranspeptidase (GGT), total bilirubin (TBIL) and total bile acid (TBA) between the two groups (*P* > 0.05). Aspartate aminotransferase (AST) was significantly decreased in the group of patients with *C. sinensis* infection (*P* < 0.05). The specific clinical biochemical parameter data for the patient has been provided in the supplementary material (S1 Table).

**Table 1:** Clinical and demographic features of patients.

	Cs group(n = 32)	Non group(n = 32)	*P*-value
Gender (Female/Male)	3/29	5/27	0.708
Age (years)	58 (52-64)	56 (49-63)	0.667
LAP (U/L)	37.5 (32.3-54.3)	44.0 (32.5-52.5)	0.554
ALT (U/L)	19.0 (15.0-42.8)	32.0 (14.3-56.5)	0.156
AST (U/L)	30.0 (24.0-53.0)	52.0 (30.3-83.3)	**0.013**
TP (g/L)	67.3 (63.1-74.5)	69.7 (65.8-75.7)	0.257
ALP (U/L)	113.5 (89.5-163.5)	103.5 (88.0-164.0)	0.605
GGT (U/L)	73.5 (34.0-170.5)	95.5 (42.3-241.8)	0.379
TBIL (μmol/L)	20.0 (14.7-57.9)	36.7 (19.8-88.0)	0.081
TBA (μmol/L)	35.8 (15.4-66.1)	50.6 (16.0-103.8)	0.448

The categorical variable (Gender) was analyzed using Fisher’s exact test. Other results were presented as median (IQR; age reported in whole years), with intergroup comparisons assessed by the Mann-Whitney U test.

### Microbial community composition analysis

**Fig 1:**
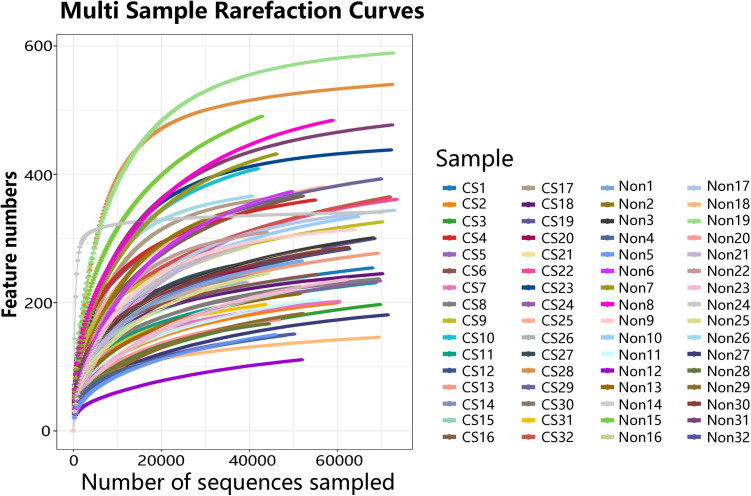
Rarefaction curves. When the curve plateaus, it indicates that the sequencing depth is adequate, and additional sequencing would yield few new species. Conversely, a non-flattening curve suggests that further sequencing may yield a higher number of new species.

A total of 3,864,697 clean reads were obtained from 64 fecal samples following filtration, assembly, and quality control procedures (S2 Table). These reads were clustered at a 97.0% similarity threshold to generate operational taxonomic units (OTUs). Taxonomic annotation of the OTUs revealed 40 phyla and 1,172 genera across all samples. The rarefaction curves indicated that the sample sizes were sufficient to capture the overall microbiota structure (**Fig 1**).

**Fig 2:**
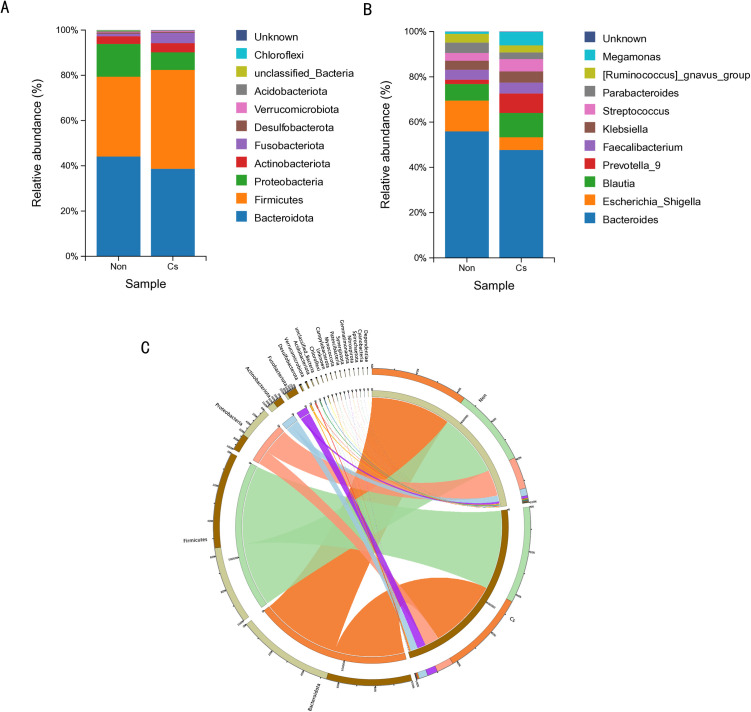
Microbial community distribution across different groups and taxonomic levels. (A) Shows the microbial community distribution at the phylum level between *C. sinensis*-infected and non-infected groups. (B) Shows the microbial community distribution at the genus level between the two groups. (C) A chord diagram of the top 20 microbial communities with the highest relative abundance at the phylum level.

To investigate the composition of gut microbiota community structures in fecal samples between the ALC concurrent with *C. sinensis* infection group and the non-infected group, this study selected the top 10 species with the highest abundance at the phylum and genus levels to construct species distribution bar charts. Analysis of **Fig 2** revealed differences in microbial composition at both phylum and genus levels between ALC patients with and without *C. sinensis* infection. At the phylum level (**Fig 2A**), Bacteroidota and Firmicutes exhibited the highest relative abundance in both groups. Firmicutes and Fusobacteriota were more abundant in the *C. sinensis*-infected group compared to the non-infected group, whereas Bacteroidota and Proteobacteria showed reduced proportions. At the genus level (**Fig 2B**), *Bacteroides* dominated both groups with the highest relative abundance. The *C. sinensis*-infected group displayed higher relative abundances of *Blautia*, *Prevotella*, *Streptococcus*, and *Megamonas* compared to the non-infected group. In contrast, *Bacteroides*, *Escherichia*, *Parabacteroides*, and *Ruminococcus_gnavus_group* were less abundant in the infected group. To further visualize the relationship between samples and microbial communities, a chord diagram (**Fig 2C**) was constructed based on the top 20 species in abundance at the phylum level. The results demonstrated that Bacteroidota, Firmicutes, and Proteobacteria constituted the dominant phyla across all samples. Notably, compared to the non-infected group, the relative abundance of Fusobacteriota was significantly increased in the *C. sinensis*-infected group. Proteobacteria were markedly reduced in the infected group. These findings suggest that Fusobacteriota and Proteobacteria may play distinct roles in the progression of *C. sinensis* infection in ALC patients.

**Fig 3:**
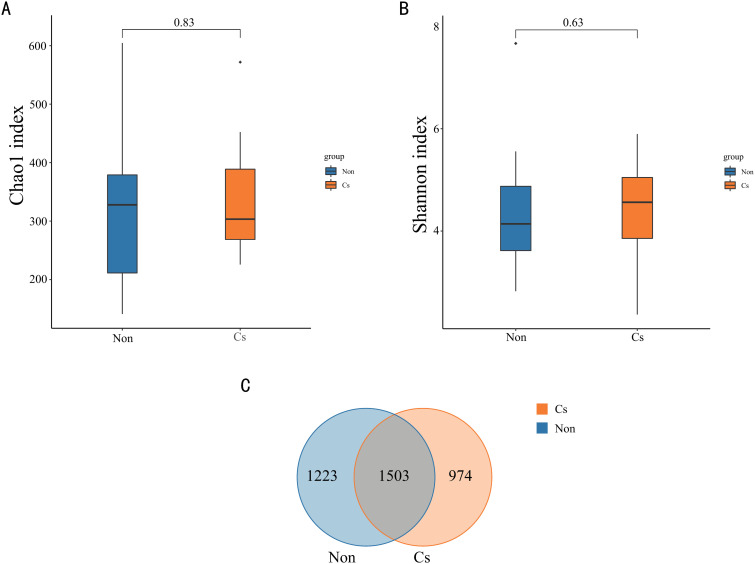
Changes in gut microbiota diversity between *C. sinensis*-infected and non-infected groups. (A) Comparison of Chao1 index between *C. sinensis*-infected and non-infected groups. (B) Comparison of Shannon index between *C. sinensis*-infected and non-infected groups. (C) Venn diagram of OTUs between the two groups.

To further assess whether there were differences in the microbial community composition between the two groups, this study employed the Mann-Whitney U test to analyze the differences at the OTU level. The α-diversity of the samples was evaluated using the Chao1 index (**Fig 3A**) and the Shannon index (**Fig 3B**). The results demonstrated that there were no significant differences in α-diversity between the microbial communities of the *C. sinensis*-infected group and the non-infected group. A Venn diagram was further utilized to visualize distinctions in gut microbiota between the two groups (**Fig 3C**). The analysis revealed that the two groups shared 1,503 OTUs out of a total of 3,700 OTUs. Notably, the number of OTUs unique to *C. sinensis*-infected patients was 974, while the number of OTUs unique to non-infected patients was 1,223. This finding indicates that compared to non-infected subjects, the clustering of OTUs in *C. sinensis*-infected patients was less diverse, suggesting that *C. sinensis* infection exerts an impact on the colonization and survival of specific gut microbiota in patients with ALC.

**Fig 4:**
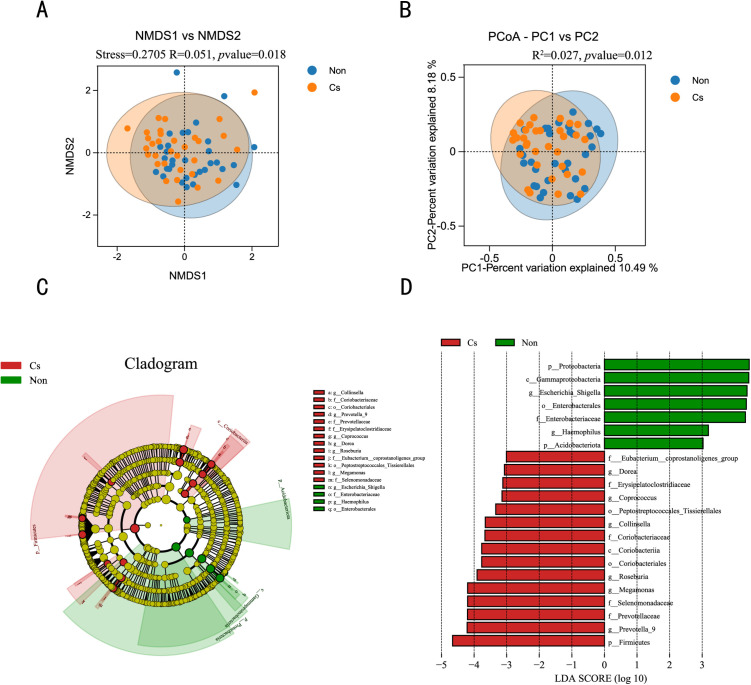
Analysis of microbial community differences between *C. sinensis*-infected and non-infected groups. (A) NMDS based on Bray-Curtis distances visualized β-diversity between two groups. ANOSIM revealed significant separation (R = 0.051, **p* *= 0.018). (B) PCoA based on Bray-Curtis distances. PERMANOVA revealed significant separation (R² = 0.027, **P* *= 0.012). (C) Cladogram from LEfSe analysis. The size of the circle diameter is proportional to the relative abundance of the taxon. (C) A histogram with linear discriminant analysis (LDA) scores based on LDA > 3.0.

Non-metric Multidimensional Scaling (NMDS) based on the Bray-Curtis distance matrix was employed to visualize the β-diversity between two groups of samples (**Fig 4A**). Although the Stress value of NMDS was 0.2705, which is near the threshold of interpretability, the ANOSIM results revealed significant differences between groups (R = 0.051, *P* = 0.018), suggesting that *C. sinensis* infection had a statistically significant effect on the gut microbiota. The potential distortion in NMDS ordination might arise from high sparsity or nonlinear structures in the data; however, the statistical significance of the test supports the authenticity of the differences between groups. To verify the robustness of the results, we further conducted Principal Coordinates Analysis (PCoA) based on the same Bray-Curtis distance matrix and PERMANOVA. The first two principal coordinates accounted for 18.67% of the variation (PC1: 10.49%, PC2: 8.18%) (**Fig 4B**). Both PCoA and NMDS showed partial overlap between the infected and non-infected groups, but the PERMANOVA results (R² = 0.027, *P* = 0.012) confirmed the biological significance of the differences between groups.

In this study, we further applied the Linear Discriminant Analysis (LDA) Effect Size (LEfSe) to identify biomarkers and characterize the differences in microbial communities between the two groups. The cladogram illustrates the phylogenetic relationships of bacterial taxa with differing relative abundances in the two groups (**Fig 4C**). Using an LDA score threshold of 3.0 and *p* < 0.05, 22 bacterial taxa showed significant differences in abundance (**Fig 4D**). In the fecal samples of non-infected patients, seven bacterial taxa exhibited significant differences, with the phylum Proteobacteriota having the highest LDA score. In patients infected with *C. sinensis*, fifteen bacterial taxa exhibited significant differences, among which the phylum Firmicutes and the genus *Prevotella* had the highest LDA scores, suggesting their potential as core gut microbiota biomarkers associated with *C. sinensis* infection.

### Intergroup correlation analysis

**Fig 5:**
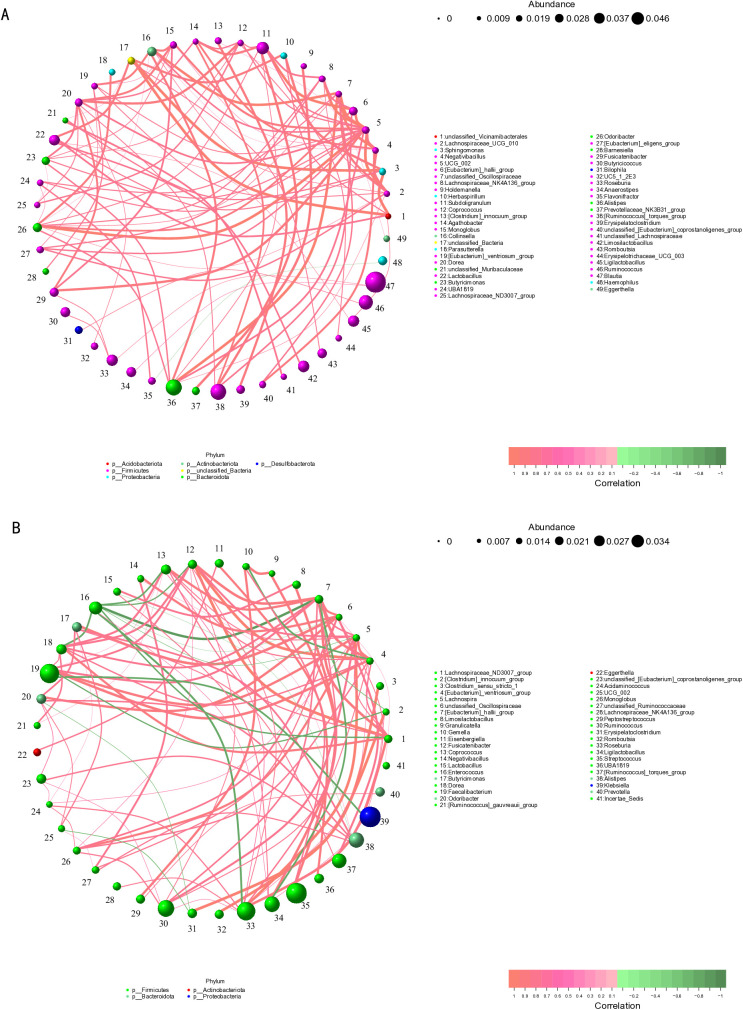
Genus-level correlation network diagrams of microbial communities in the two groups. (A) Correlation network diagram of the non-infected group. (B) Correlation network diagram of the *C. sinensis*-infected group. In the figure, nodes represent genera, with node size indicating relative abundance and node color representing different phylum-level classifications. Edges represent significant genus-level correlations (red: positive, green: negative), and the thickness of edges reflects the strength of correlations.

To elucidate the interactions among microbial communities, we constructed genus-level correlation networks using Spearman’s rank correlation coefficient (threshold: |ρ| > 0.1 and *P* < 0.05) (**Fig 5**). Nodes represent bacterial genera, while edges denote significant inter-genus correlations (red: positive, green: negative). Topological analysis revealed that the network of the non-infected group exhibited a typical structure dominated by positive correlations, with positive edges accounting for 99.0% of total connections, uniformly distributed edges, and a relatively low network density (0.085) (**Fig 5A**). Four keystone hub genera were identified in the non-infected group: *Ruminococcaceae_UCG-002*, *Odoribacter*, *Dorea*, and *Lachnospiraceae_UCG-010*. In stark contrast, the network topology of the *C. sinensis*-infected group showed increased complexity, with the network density rising to 0.122 and the proportion of negative correlation edges increasing to 16.0% (**Fig 5B**). *Enterococcus* exhibited strong negative correlations with multiple nodes. *Enterococcus*, *Eubacterium_ventriosum_group*, *Lachnospira*, and *Eubacterium_hallii_group* formed a new core interaction module. Phylum-level classification showed that the core genera in the non-infected group were distributed across seven different phyla, whereas the core genera in the *C. sinensis*-infected group were limited to four phyla. These significant differences in network topology suggest that *C. sinensis* infection may restructure microbial interaction networks, leading to reduced community stability. These findings offer new insights into the impact of *C. sinensis* infection on alterations in gut microbial communities.

### Functional prediction of microbial communities

**Fig 6:**
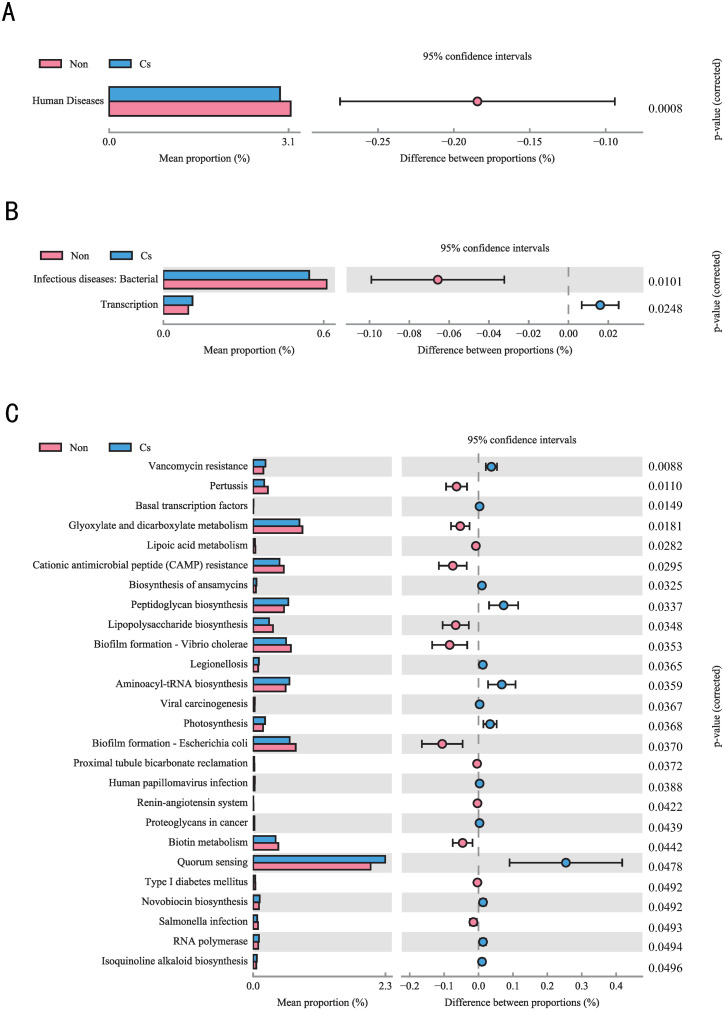
KEGG pathway analysis of microbial functional profiles predicted by Tax4Fun2. (A) Significantly differential KEGG Level 1 pathways; (B) Significantly differential KEGG Level 2 pathways; (C) Significantly differential KEGG Level 3 pathways. The left side shows the mean proportion of different functions in the two sample groups. The middle section indicates the difference in proportion of functions within the 95% confidence interval. The values on the far right are *P*-values (*P* < 0.05).

To deeply explore the functional differences in gut microbiota between *C. sinensis*-infected and non-infected groups, this study employed the R package Tax4Fun2, based on the SILVA database, to predict microbial community functions. [32] (**Fig 6**). In KEGG Level 1 classification, only the “Human Diseases” functional pathway showed significant differences between the infected and control groups (*P* < 0.05) (**Fig 6A**). KEGG Level 2 pathway analysis further found that the “Infectious Diseases: Bacterial” and “Transcription” functional modules exhibited significant differences between the two groups (*P* < 0.05) (**Fig 6B**). Through KEGG Level 3 pathway analysis, we observed significant functional divergence between the two groups (**Fig 6C**). In the *C. sinensis*-infected group, pathways related to pathogen adaptation were significantly enriched, including resistance pathways (Vancomycin resistance), microbial structural synthesis pathways (Peptidoglycan biosynthesis), and pathogen-host interaction pathways (Legionellosis, Human papillomavirus infection). In contrast, the non-infected group primarily enriched pathways related to host metabolic regulation (Glyoxylate and dicarboxylate metabolism, Lipoic acid metabolism), and physiological homeostasis (Renin-angiotensin system, Proximal tubule bicarbonate reclamation). Notably, the Vancomycin resistance pathway showed the strongest differential signal in the infected group (**P* *= 0.0088). These multi-level analytical results demonstrate that *C. sinensis* infection significantly remodels the functional network architecture of the gut microbiota in patients with ALC (*P* < 0.05).

## Discussion

Numerous studies have demonstrated that individuals with low socioeconomic status, limited educational attainment, and reduced income face a significantly heightened risk of developing ALD [33]. In economically underdeveloped regions such as China and Southeast Asia, the dual threat of *C. sinensis* infection and ALD poses significant health risks to local residents, necessitating urgent attention. This study employed 16S rRNA gene sequencing technology to conduct a preliminary investigation into the relationship between *C. sinensis* infection and the gut microbiota of patients suffering from ALC. To our knowledge, this represents the first study to explore the gut microbiota in patients with ALC and *C. sinensis* infection.

Our study demonstrated that assessments of α-diversity in bacterial communities, utilizing the Chao1 and Shannon indices, revealed no significant differences between the *C. sinensis*-infected and non-infected groups. This finding is consistent with previous research on the gut microbiota of patients with parasitic infections [27,34,35]. This study employed both NMDS and PCoA analyses to assess the β-diversity between the two groups. The results demonstrated that the microbial community in the *C. sinensis*-infected group exhibited significant differences compared to the non-infected group, suggesting that *C. sinensis* infection may modify the gut microbiota composition in patients with ALC. The LEfSe analysis demonstrated that, relative to the non-infected group, the *C. sinensis*-infected group displayed significant intergroup divergence across multiple bacterial phyla and genera, with Firmicutes exhibiting the most substantial contribution to phylum-level disparities, whereas the genus *Prevotella* emerged as the predominant contributor at the genus level. These taxa (Firmicutes and *Prevotella*) were established as pivotal discriminative taxa underpinning the gut microbiota compositional disparities in ALC patients with *C. sinensis* infection. Firmicutes releases immunologically active cell wall glycoconjugates under the action of intestinal lysozyme. These glycoconjugates activate interleukin-34 (IL-34), thereby enhancing systemic immune defense [36]. Firmicutes, as a key taxon in the infected group, suggests its potential role in the immune response triggered by *C. sinensis* infection in humans. *Prevotella* is a diverse genus of Gram-negative anaerobic bacteria. While *Prevotella* contains no known obligate pathogenic species, yet members have been implicated in multiple diseases, including inflammatory autoimmune diseases, opportunistic infections, bacterial vaginosis, oral biofilm formation, and biofilm-associated oral diseases [37,38]. However, current reports remain contradictory as to whether *Prevotella* exerts beneficial or detrimental effects on human health [37,39,40]. We contend that the relative enrichment of *Prevotella* in the *C. sinensis*-infected group may be associated with the proinflammatory state in the gut and liver of patients with ALC. It has been confirmed by studies that alcohol disrupts the gut-liver axis at multiple interconnected levels, including the gut microbiome, mucus barrier, epithelial barrier and at the level of antimicrobial peptide production, which increases microbial exposure and the proinflammatory environment of the liver [41,42]. Furthermore, studies have shown that *Prevotella* activates Toll-like receptor 2, leading to production of Th17-polarizing cytokines by antigen-presenting cells, including IL-23 and IL-1. Concurrently, *Prevotella* stimulate epithelial cells to produce IL-8, IL-6 and CCL20, which can promote mucosal Th17 immune responses and neutrophil recruitment, triggering local inflammation [43]. This sustained inflammatory tone may further exacerbate gut-liver axis dysfunction, promote bacterial translocation, and aggravate liver inflammation. Therefore, in the context of ALC, the enrichment of *Prevotella* may not only serve as a biomarker but also act as an active driver of disease progression, exerting significant proinflammatory potential in the gut and liver of affected patients.

Notably, correlation analyses at the genus level of the microbial community revealed distinct differences between the *C. sinensis*-infected and non-infected groups. In the *C. sinensis*-infected group, *Enterococcus* exhibited significant negative correlations with multiple other genera, a phenomenon not observed in the non-infected group. As commensals, *Enterococci* colonize the digestive system and participate in the modulation of the immune system in humans and animals [44]. Studies have demonstrated that bacteriocins produced by enterococci can modulate niche competition between enterococci and the intestinal microbiota, thereby influencing the microecological network and effectively combating a range of both Gram-positive and Gram-negative bacteria, including *Acinetobacter*, *Bacillus*, *Clostridium*, *Klebsiella*, *Lactobacillus*, and *Pseudomonas* [45–47]. This study demonstrates that *C. sinensis* infection alters the intestinal microbiota balance in patients with ALC, thereby affecting the symbiotic relationships among different microbial communities and causing *Enterococcus* to exhibit a significant negative correlation within the gut microecology. Inter-group difference analysis based on KEGG pathway enrichment indicates that vancomycin resistance is the most significantly different level 3 pathway among numerous divergent pathways between the two groups. Numerous reports indicate that infections caused by vancomycin-resistant bacteria, such as vancomycin-resistant *Enterococcus* (VRE) and vancomycin-resistant *Staphylococcus aureus* (VRSA), significantly impact the mortality of cirrhosis patients [48–50]. Considering the changes in *Enterococcus* within the gut microecology of the two groups in this study, as well as the differences in KEGG pathway enrichment for vancomycin resistance, we propose that *C. sinensis* infection may exacerbate the disease burden in patients with ALC. However, this perspective requires further confirmation through additional basic research.

Notably, this study has certain limitations. First, this is a single-center study limited to the population with the same ethnicity and dietary habits in the Pearl River Delta region of China, with a small sample size. Thus, it may not represent the overall gut microbiota changes in all ALC patients with *C. sinensis* infection. Our results need further confirmation by a large-scale, multi-center cohort study. Second, though strict inclusion and exclusion criteria for participants were set, different disease progression stages and liver injury degrees might affect the gut microbiota. Lastly, our experimental design did not include a control group of healthy adults without liver disease or *C. sinensis* infection. The microbial characteristics observed in the ALC with *C. sinensis* infection group may reflect superimposed or synergistic effects. In the next phase of the study, we plan to incorporate additional control groups to specifically dissect the independent effects of *C. sinensis* infection and ALC. Despite these limitations, we used next-generation sequencing on specific genomic regions of the microbiome to obtain reliable data, which supports the role of *C. sinensis* infection in changing the gut microbiota of ALC patients. Our study lays a foundation for the diagnosis, treatment, and future large-scale research in this field.

## Conclusion

Overall, this study reveals the impact of *C. sinensis* infection on the gut microbiota of patients suffering from ALC in the Pearl River Delta region of China. Patients with ALC and *C. sinensis* infection show alterations in gut microbiota compared to non-infected counterparts. Notably, specific bacteria such as *Prevotella* and *Enterococcus* may represent potential targets for the development of novel diagnostic and therapeutic strategies for ALC patients afflicted by *C. sinensis* infection. Nevertheless, further research is imperative before these findings can be translated into clinical applications.

## Supporting information

S1 Table Demographic and clinical biochemical parameters data of patients. (DOCX)

S2 Table Statistical summary of sample sequencing data. (DOCX)
