# Supplementary material for: Alterations in the gut microbiota of alcoholic cirrhosis patients infected with Clonorchis sinensis in the Pearl River Delta region of China
Source: PLoS One. 2025 Oct 21;20(10):e0334311. doi: 10.1371/journal.pone.0334311 (PMC12539714; doi:10.1371/journal.pone.0334311)
Supplement: S1 Table — (DOCX) [file pone.0334311.s001.docx]

**Supporting information**

**S1 Table. Demographic and clinical biochemical parameters data of patients.**

| **Sample ID** | **Gender** | **Age (years)** | **LAP (U/L)** | **ALT (U/L)** | **AST (U/L)** | **TP (g/L)** | **ALP (U/L)** | **GGT (U/L)** | **TBIL (μmol/L)** | **TBA (μmol/L)** |
| --- | --- | --- | --- | --- | --- | --- | --- | --- | --- | --- |
| Cs1 | Male | 52 | 35 | 36 | 32 | 81 | 98 | 62 | 10.9 | 9.5 |
| Cs2 | Male | 63 | 25 | 7 | 24 | 65 | 81 | 21 | 69.2 | 27.6 |
| Cs3 | Male | 44 | 37 | 15 | 27 | 62.6 | 151 | 25 | 51.7 | 152.1 |
| Cs4 | Male | 62 | 31 | 20 | 29 | 64.2 | 109 | 47 | 69.7 | 83 |
| Cs5 | Male | 49 | 36 | 15 | 15 | 77.3 | 159 | 59 | 19 | 5.2 |
| Cs6 | Male | 46 | 37 | 15 | 23 | 65.6 | 180 | 78 | 19.8 | 26 |
| Cs7 | Male | 62 | 66 | 61 | 68 | 65 | 165 | 133 | 14 | 13.5 |
| Cs8 | Female | 55 | 67 | 45 | 25 | 76.7 | 248 | 538 | 40.6 | 19.8 |
| Cs9 | Male | 52 | 60 | 8 | 71 | 72.9 | 335 | 343 | 59.9 | 77.3 |
| Cs10 | Male | 58 | 25 | 17 | 53 | 55.7 | 141 | 87 | 10.2 | 42.8 |
| Cs11 | Male | 70 | 38 | 13 | 29 | 67.6 | 139 | 174 | 24.4 | 2.6 |
| Cs12 | Female | 21 | 47 | 16 | 55 | 66.9 | 83 | 63 | 349.4 | 281 |
| Cs13 | Male | 51 | 24 | 11 | 30 | 61.3 | 96 | 69 | 62.8 | 71.1 |
| Cs14 | Male | 64 | 32 | 24 | 32 | 82.4 | 113 | 34 | 16.4 | 16.4 |
| Cs15 | Male | 65 | 33 | 15 | 18 | 83 | 194 | 66 | 12.9 | 40.7 |
| Cs16 | Male | 53 | 39 | 20 | 28 | 59.9 | 91 | 21 | 122.1 | 359.5 |
| Cs17 | Male | 35 | 55 | 46 | 72 | 78.9 | 76 | 719 | 65.6 | 11.2 |
| Cs18 | Male | 65 | 36 | 23 | 26 | 65.8 | 104 | 129 | 16.2 | 27.4 |
| Cs19 | Male | 58 | 92 | 37 | 49 | 66.3 | 255 | 425 | 23.2 | 4.3 |
| Cs20 | Male | 55 | 39 | 15 | 24 | 62.6 | 89 | 23 | 12.6 | 35.2 |
| Cs21 | Male | 66 | 73 | 52 | 48 | 71.8 | 261 | 175 | 17 | 36.3 |
| Cs22 | Male | 60 | 52 | 18 | 24 | 71.9 | 114 | 138 | 18.7 | 40.2 |
| Cs23 | Male | 63 | 62 | 43 | 78 | 75.3 | 87 | 625 | 40.9 | 15.1 |
| Cs24 | Male | 53 | 21 | 9 | 16 | 70.4 | 53 | 30 | 20.6 | 36.7 |
| Cs25 | Male | 52 | 40 | 82 | 76 | 74.5 | 60 | 197 | 13.5 | 5.5 |
| Cs26 | Female | 64 | 32 | 42 | 30 | 74.4 | 132 | 119 | 20.1 | 26 |
| Cs27 | Male | 58 | 56 | 99 | 155 | 68.1 | 191 | 84 | 28.5 | 123.1 |
| Cs28 | Male | 69 | 38 | 15 | 21 | 63 | 114 | 160 | 19.4 | 49.6 |
| Cs29 | Male | 66 | 33 | 11 | 22 | 69.2 | 78 | 21 | 14.2 | 27.5 |
| Cs30 | Male | 64 | 48 | 17 | 36 | 63.3 | 146 | 50 | 7.6 | 37.4 |
| Cs31 | Male | 52 | 32 | 49 | 53 | 58.5 | 109 | 34 | 18.6 | 51.2 |
| Cs32 | Male | 64 | 34 | 20 | 41 | 57.9 | 95 | 10 | 237.2 | 246.3 |
| Non1 | Male | 58 | 67 | 117 | 251 | 65.5 | 169 | 296 | 52.7 | 26.8 |
| Non2 | Male | 65 | 53 | 13 | 26 | 66.8 | 92 | 51 | 38 | 30.6 |
| Non3 | Female | 62 | 61 | 22 | 38 | 67.2 | 143 | 327 | 33.8 | 54.5 |
| Non4 | Male | 47 | 46 | 18 | 36 | 73.4 | 202 | 97 | 35.3 | 105.5 |
| Non5 | Male | 38 | 50 | 32 | 65 | 68.6 | 265 | 63 | 104.8 | 125.7 |
| Non6 | Male | 61 | 48 | 30 | 34 | 68.2 | 134 | 146 | 27.2 | 49.5 |
| Non7 | Female | 67 | 34 | 70 | 27 | 64.4 | 66 | 65 | 15.1 | 2 |
| **Sample ID** | **Gender** | **Age (years)** | **LAP (U/L)** | **ALT (U/L)** | **AST (U/L)** | **TP (g/L)** | **ALP (U/L)** | **GGT (U/L)** | **TBIL (μmol/L)** | **TBA (μmol/L)** |
| Non8 | Male | 49 | 45 | 7 | 44 | 81.4 | 115 | 91 | 92.2 | 344 |
| Non9 | Male | 52 | 44 | 40 | 72 | 70.2 | 96 | 134 | 49 | 31.5 |
| Non10 | Male | 68 | 60 | 36 | 61 | 94.9 | 91 | 40 | 87.9 | 381.8 |
| Non11 | Male | 56 | 27 | 10 | 26 | 58.2 | 250 | 16 | 107 | 487.7 |
| Non12 | Male | 33 | 101 | 44 | 157 | 82.9 | 118 | 1804 | 44.3 | 63.2 |
| Non13 | Male | 41 | 26 | 18 | 22 | 70 | 47 | 49 | 17.4 | 3.8 |
| Non14 | Male | 56 | 44 | 32 | 60 | 87.3 | 95 | 36 | 98.4 | 111.4 |
| Non15 | Male | 82 | 32 | 55 | 53 | 65.5 | 87 | 67 | 10.7 | 6.7 |
| Non16 | Male | 55 | 30 | 20 | 34 | 78 | 100 | 38 | 27.5 | 21 |
| Non17 | Female | 61 | 32 | 12 | 30 | 76.5 | 104 | 28 | 19.8 | 98.8 |
| Non18 | Male | 59 | 20 | 11 | 13 | 70.5 | 95 | 30 | 12 | 2.2 |
| Non19 | Male | 74 | 44 | 69 | 92 | 70.3 | 99 | 94 | 56.5 | 61.8 |
| Non20 | Male | 54 | 37 | 102 | 303 | 68.5 | 71 | 196 | 44.1 | 84.2 |
| Non21 | Male | 63 | 24 | 12 | 26 | 66.7 | 65 | 25 | 19.8 | 16.5 |
| Non22 | Female | 55 | 91 | 25 | 99 | 69.4 | 284 | 927 | 88 | 109.1 |
| Non23 | Male | 69 | 34 | 57 | 57 | 70.2 | 103 | 151 | 3.7 | 15.8 |
| Non24 | Male | 63 | 34 | 12 | 31 | 63.1 | 113 | 24 | 132.8 | 464.3 |
| Non25 | Male | 46 | 149 | 84 | 221 | 71.4 | 349 | 2276 | 77.9 | 52.8 |
| Non26 | Male | 50 | 79 | 92 | 188 | 70 | 105 | 966 | 20.7 | 8.5 |
| Non27 | Female | 54 | 30 | 22 | 34 | 61.1 | 50 | 128 | 16.2 | 10.9 |
| Non28 | Male | 54 | 45 | 33 | 29 | 77.3 | 82 | 177 | 15.3 | 3.1 |
| Non29 | Male | 49 | 35 | 13 | 55 | 67.4 | 192 | 261 | 91.2 | 51.7 |
| Non30 | Male | 53 | 49 | 35 | 51 | 62.2 | 149 | 116 | 28.5 | 35.1 |
| Non31 | Male | 69 | 38 | 63 | 87 | 65.2 | 85 | 257 | 94.4 | 27.4 |
| Non32 | Male | 45 | 51 | 50 | 67 | 84.7 | 171 | 58 | 29.8 | 71.8 |

Abbreviations: LAP, leucine aminopeptidase; ALT, alanine aminotransferase; AST, aspartate aminotransferase; TP, total protein; ALP, alkaline phosphatase; GGT, γ-glutamyltranspeptidase; TBIL, total bilirubin; TBA, total bile acid.
