# Supplementary material for: Alterations in the gut microbiota of alcoholic cirrhosis patients infected with Clonorchis sinensis in the Pearl River Delta region of China
Source: PLoS One. 2025 Oct 21;20(10):e0334311. doi: 10.1371/journal.pone.0334311 (PMC12539714; doi:10.1371/journal.pone.0334311)
Supplement: S2 Table — (DOCX) [file pone.0334311.s002.docx]

**S2 Table.** **Statistical Summary of Sample Sequencing Data.**

| **Sample ID** | **Raw Reads** | **Clean Reads** | **Effective Reads** | **AvgLen(bp)** | **GC(%)** | **Q20(%)** | **Q30(%)** | **Effective(%)** |
| --- | --- | --- | --- | --- | --- | --- | --- | --- |
| Cs1 | 80044 | 75425 | 69364 | 416 | 52.42 | 99.14 | 96.47 | 86.66 |
| Cs2 | 65994 | 62039 | 60505 | 420 | 49.42 | 99.16 | 96.41 | 91.68 |
| Cs3 | 80075 | 74866 | 71378 | 413 | 53.23 | 99.12 | 96.38 | 89.14 |
| Cs4 | 67009 | 63027 | 58134 | 421 | 52.76 | 99.14 | 96.42 | 86.76 |
| Cs5 | 56302 | 52754 | 51174 | 416 | 50.82 | 99.10 | 96.31 | 90.89 |
| Cs6 | 65821 | 61490 | 57180 | 418 | 51.32 | 99.11 | 96.32 | 86.87 |
| Cs7 | 68316 | 63834 | 61212 | 418 | 50.21 | 99.06 | 96.18 | 89.60 |
| Cs8 | 80047 | 74778 | 70267 | 417 | 50.35 | 99.13 | 96.36 | 87.78 |
| Cs9 | 79966 | 74873 | 71843 | 417 | 51.69 | 99.13 | 96.45 | 89.84 |
| Cs10 | 46000 | 43083 | 42472 | 422 | 51.45 | 99.07 | 96.21 | 92.33 |
| Cs11 | 79581 | 74192 | 69948 | 417 | 50.05 | 99.10 | 96.26 | 87.90 |
| Cs12 | 71874 | 67171 | 64807 | 423 | 51.65 | 99.08 | 96.27 | 90.17 |
| Cs13 | 79872 | 74983 | 71110 | 421 | 51.84 | 99.08 | 96.26 | 89.03 |
| Cs14 | 49995 | 46760 | 43512 | 417 | 50.47 | 99.12 | 96.40 | 87.03 |
| Cs15 | 57147 | 53645 | 52856 | 415 | 52.40 | 99.18 | 96.56 | 92.49 |
| Cs16 | 57959 | 54118 | 52743 | 421 | 51.49 | 99.07 | 96.21 | 91.00 |
| Cs17 | 53330 | 49516 | 48927 | 426 | 50.46 | 99.03 | 96.05 | 91.74 |
| Cs18 | 80012 | 74988 | 72025 | 417 | 50.30 | 99.17 | 96.48 | 90.02 |
| Cs19 | 79983 | 75251 | 70199 | 418 | 53.28 | 99.15 | 96.48 | 87.77 |
| Cs20 | 50692 | 46988 | 43926 | 419 | 52.14 | 98.93 | 95.75 | 86.65 |
| Cs21 | 65688 | 61334 | 57979 | 419 | 52.56 | 99.12 | 96.41 | 88.26 |
| Cs22 | 80004 | 75333 | 74011 | 418 | 52.51 | 99.11 | 96.35 | 92.51 |
| Cs23 | 79745 | 73274 | 72633 | 425 | 53.76 | 99.03 | 96.06 | 91.08 |
| Cs24 | 77657 | 72933 | 71088 | 417 | 52.26 | 99.18 | 96.56 | 91.54 |
| Cs25 | 62691 | 59002 | 57593 | 415 | 51.89 | 99.19 | 96.59 | 91.87 |
| Cs26 | 43652 | 40724 | 39760 | 422 | 50.10 | 99.08 | 96.24 | 91.08 |
| Cs27 | 40231 | 37804 | 37567 | 423 | 54.02 | 99.16 | 96.50 | 93.38 |
| Cs28 | 79952 | 73895 | 73173 | 409 | 49.82 | 99.22 | 96.62 | 91.52 |
| Cs29 | 80002 | 74956 | 71873 | 414 | 52.16 | 99.15 | 96.51 | 89.84 |
| Cs30 | 58984 | 55556 | 53278 | 420 | 52.36 | 99.18 | 96.55 | 90.33 |
| Cs31 | 49575 | 46126 | 44306 | 421 | 50.05 | 99.07 | 96.18 | 89.37 |
| Cs32 | 79691 | 75103 | 73069 | 416 | 51.54 | 99.16 | 96.49 | 91.69 |
| Non1 | 58431 | 54751 | 52593 | 422 | 49.91 | 99.09 | 96.22 | 90.01 |
| Non2 | 40454 | 37929 | 37444 | 420 | 51.99 | 99.05 | 96.14 | 92.56 |
| Non3 | 75086 | 70671 | 68964 | 418 | 53.43 | 99.18 | 96.57 | 91.85 |
| Non4 | 55878 | 52258 | 49211 | 422 | 49.36 | 99.05 | 96.10 | 88.07 |
| Non5 | 50863 | 47727 | 46301 | 423 | 54.70 | 99.11 | 96.34 | 91.03 |
| Non6 | 54459 | 51210 | 50139 | 422 | 49.58 | 99.06 | 96.13 | 92.07 |
| Non7 | 51588 | 48193 | 46988 | 417 | 51.22 | 99.05 | 96.16 | 91.08 |
| Non8 | 64146 | 60006 | 59475 | 414 | 53.45 | 99.15 | 96.49 | 92.72 |
| Non9 | 69900 | 65559 | 64464 | 423 | 49.02 | 99.09 | 96.24 | 92.22 |
| **Sample ID** | **Raw Reads** | **Clean Reads** | **Effective Reads** | **AvgLen(bp)** | **GC(%)** | **Q20(%)** | **Q30(%)** | **Effective(%)** |
| Non10 | 72656 | 67771 | 65594 | 419 | 50.13 | 99.03 | 96.05 | 90.28 |
| Non11 | 66696 | 62652 | 57415 | 422 | 50.00 | 99.05 | 96.13 | 86.08 |
| Non12 | 62056 | 58041 | 52921 | 421 | 49.40 | 99.13 | 96.41 | 85.28 |
| Non13 | 59595 | 56148 | 52480 | 421 | 49.54 | 99.10 | 96.25 | 88.06 |
| Non14 | 57551 | 53816 | 51517 | 420 | 49.74 | 99.09 | 96.20 | 89.52 |
| Non15 | 47234 | 44410 | 43634 | 419 | 53.22 | 99.16 | 96.54 | 92.38 |
| Non16 | 43289 | 40753 | 39770 | 417 | 50.81 | 99.11 | 96.30 | 91.87 |
| Non17 | 80012 | 74013 | 73450 | 418 | 52.53 | 99.08 | 96.21 | 91.80 |
| Non18 | 80020 | 75326 | 71944 | 421 | 50.91 | 99.13 | 96.38 | 89.91 |
| Non19 | 80143 | 74163 | 73337 | 419 | 52.13 | 99.07 | 96.20 | 91.51 |
| Non20 | 52223 | 49072 | 47139 | 417 | 50.34 | 99.15 | 96.46 | 90.26 |
| Non21 | 46920 | 43920 | 42735 | 424 | 49.93 | 99.09 | 96.24 | 91.08 |
| Non22 | 48125 | 45310 | 44353 | 417 | 52.22 | 99.16 | 96.49 | 92.16 |
| Non23 | 79992 | 74919 | 63486 | 422 | 51.25 | 99.06 | 96.19 | 79.37 |
| Non24 | 79957 | 75303 | 71471 | 420 | 54.48 | 99.10 | 96.33 | 89.39 |
| Non25 | 49537 | 46146 | 44660 | 422 | 52.18 | 99.05 | 96.13 | 90.15 |
| Non26 | 44026 | 41342 | 40927 | 416 | 52.33 | 99.15 | 96.49 | 92.96 |
| Non27 | 79968 | 75597 | 72590 | 420 | 52.29 | 99.17 | 96.51 | 90.77 |
| Non28 | 48878 | 46021 | 44938 | 418 | 51.35 | 99.14 | 96.38 | 91.94 |
| Non29 | 57812 | 54108 | 52818 | 423 | 50.19 | 99.10 | 96.25 | 91.36 |
| Non30 | 77580 | 72967 | 65725 | 421 | 51.12 | 99.11 | 96.35 | 84.72 |
| Non31 | 80081 | 74427 | 72983 | 420 | 50.60 | 99.06 | 96.14 | 91.14 |
| Non32 | 64487 | 60347 | 50593 | 420 | 51.10 | 99.10 | 96.25 | 78.45 |

Sample ID refers to the sample identifier. Raw Reads refer to the total number of original reads obtained from sequencing. Clean Reads denote the number of high-quality reads that are derived from the quality control of the original sequences. Effective Reads represent the valid sequences obtained after the assembly (paired-end), length filtering, and chimera removal from the Clean Reads. AvgLen (bp) indicates the average sequence length of the sample. GC (%) represents the GC content of the sample, which is the percentage of guanine (G) and cytosine (C) bases relative to the total number of bases. Q20 (%) denotes the percentage of bases with a quality value greater than or equal to 20 among the total bases. Q30 (%) indicates the percentage of bases with a quality value greater than or equal to 30 among the total bases. Effective (%) represents the percentage of Effective Reads in relation to Raw Reads.
